# Extreme thermal environments: reservoirs of industrially important thermozymes

**DOI:** 10.3389/fmicb.2025.1739143

**Published:** 2026-01-09

**Authors:** Anita Pandey, Kusum Dhakar

**Affiliations:** 1 Department of Biotechnology, Graphic Era (Deemed to be University), Dehradun, Uttarakhand, India; 2 Department of Aquatic Microbial Ecology, Institute of Hydrobiology, Biology Centre CAS, Ceske Budejovice, Czechia

**Keywords:** conservation, cultivation, industrial applications, phylogeny, thermal environments, thermophiles

## Abstract

Extreme thermal environments, both natural (e.g., hot springs, fumaroles, geysers, mud pots, deep-sea hydrothermal vents) and man-made (e.g., compost heaps, sawdust, coal refuse piles), are rich sources of thermophilic microorganisms, including Bacteria and Archaea. These organisms possess unique adaptations that allow survival and metabolic activity at elevated temperatures, making them valuable sources of thermostable and thermoactive enzymes. This review synthesizes current knowledge on thermophiles, including their phylogeny, adaptation mechanisms, and cultivation strategies. We discuss the industrial applications of thermozymes, such as DNA polymerases and other thermostable enzymes, and highlight the role of genomics, systems biology, and bioinformatics in accelerating enzyme discovery. The review also addresses the astrobiological relevance of thermophiles as models for life in extreme extraterrestrial environments and emphasizes the importance of conservation and sustainable use of natural thermal habitats. Collectively, this overview provides a comprehensive perspective on the ecological, biotechnological, and fundamental research significance of thermophiles and their enzymes.

## 1 Introduction

**Figure 1:**
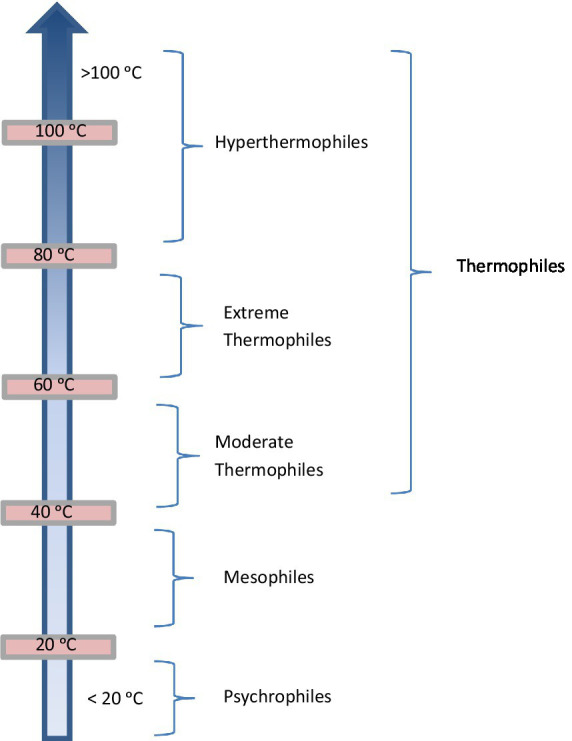
Classification of microorganisms based on temperature (Source: Brock, 1985; Blöchl et al., 1997; Stetter, 2006).

Microorganisms, based on their temperature requirements, may be classified as psychrophiles (−2 to 20 °C), mesophiles (20–40 °C), and thermophiles and hyperthermophiles (45–113 °C) (Figure 1). While the upper limit for eukaryotic life is ~60 °C, several groups of microorganisms can cope with the stress of high temperature. In general, microorganisms with optimal growth temperatures between 60° and 108 °C are referred to as thermophiles (Tansey and Brock, 1972; Hickey and Singer, 2004). Further, thermophilic microorganisms may be sub-classified as thermophiles: primarily Bacteria that display optimal growth temperature between 60° and 80 °C, and hyperthermophiles: primarily Archaea that grow optimally at 80 °C or above, being unable to grow below 60 °C. Thermophilic microorganisms are generally isolated from several marine and terrestrial geothermally heated habitats (Wang et al., 2015).

Thomas D. Brock, for the first time, reported the existence of microorganisms from the boiling hot springs of Yellowstone National Park (Brock and Freeze, 1969). Since then, a variety of microorganisms adapted to high temperatures have been discovered. These include phototrophs that grow up to 72–73 °C and heterotrophs that grow up to 91 °C (the temperature at which water boils at the elevation of Yellowstone). In 1996, an extraordinary microorganism, *Pyrolobus fumarii*, from a deep-sea hydrothermal vent was isolated, that was capable of growing up to 113 °C, and was unable to grow below 90 °C (Blöchl et al., 1997). This discovery led to naming a category of thermophiles as hyperthermophiles that grow at 80 °C or above. Since then, over 90 hyperthermophilic species have been discovered, mostly belonging to the domain of Archaea, and some to Bacteria. Throughout evolution, the thermophilic organisms were able to adapt to changing environments, such as, the up-shifts in temperature. The current classification considers thermophiles—all organisms growing above 55 °C, moderate thermophiles—organisms growing above 65 °C, extreme thermophiles—organisms growing above 75 °C, and hyperthermophiles—organisms growing above 90 °C (Imanaka, 2011). Some authors refer hyperthermophiles that include extreme thermophiles as well (Blöchl et al., 1997; Stetter, 2006). Since Brock’s discovery, thermophiles have been discovered from a variety of geothermal areas all over the world, including areas in North America, Iceland, New Zealand, Japan, Italy, India, China, Tibet, Turkey, Tunisia and the Soviet Union.^1^

Thermophilic microorganisms are particularly fascinating due to their structural and physiological features allowing them to withstand extremely selective environmental conditions. These properties are often due to specific biomolecules (DNA, lipids, enzymes, osmolites, etc.) that have been studied for years as novel sources for biotechnological and industrial applications (Elleuche et al., 2014). In late 1980s, the first hyperthermophilic enzymes were purified, that proved to be extremely stable at high temperatures (Vieille and Zeikus, 2001; Unsworth et al., 2007). It was also found that these enzymes can be cloned and expressed in mesophilic hosts without losing their active conformation and thermostability. The findings on increasing number of thermostable enzymes from thermophiles opened new possibilities of commercial relevance mainly in starch industry, synthesis of amino acids, petroleum, chemical, pulp and paper industries, and in many other fields (Zeldes et al., 2015). The emerging eco-friendly industrial processes are receiving attention in view of implementing the bio-based technologies and reshaping the biocatalytic functions (Turner et al., 2007; Zimmermann, 2025).

In the present review, we synthesize recent advances in thermophile-derived enzymes (“thermozymes”), highlight the industrial applications supported by their exceptional thermostability, and provide an updated, application-oriented overview of their emerging and established roles in industrial enzyme discovery, along with insights into the conservation of such extreme habitats.

## 2 Thermal environments

**Figure 2:**
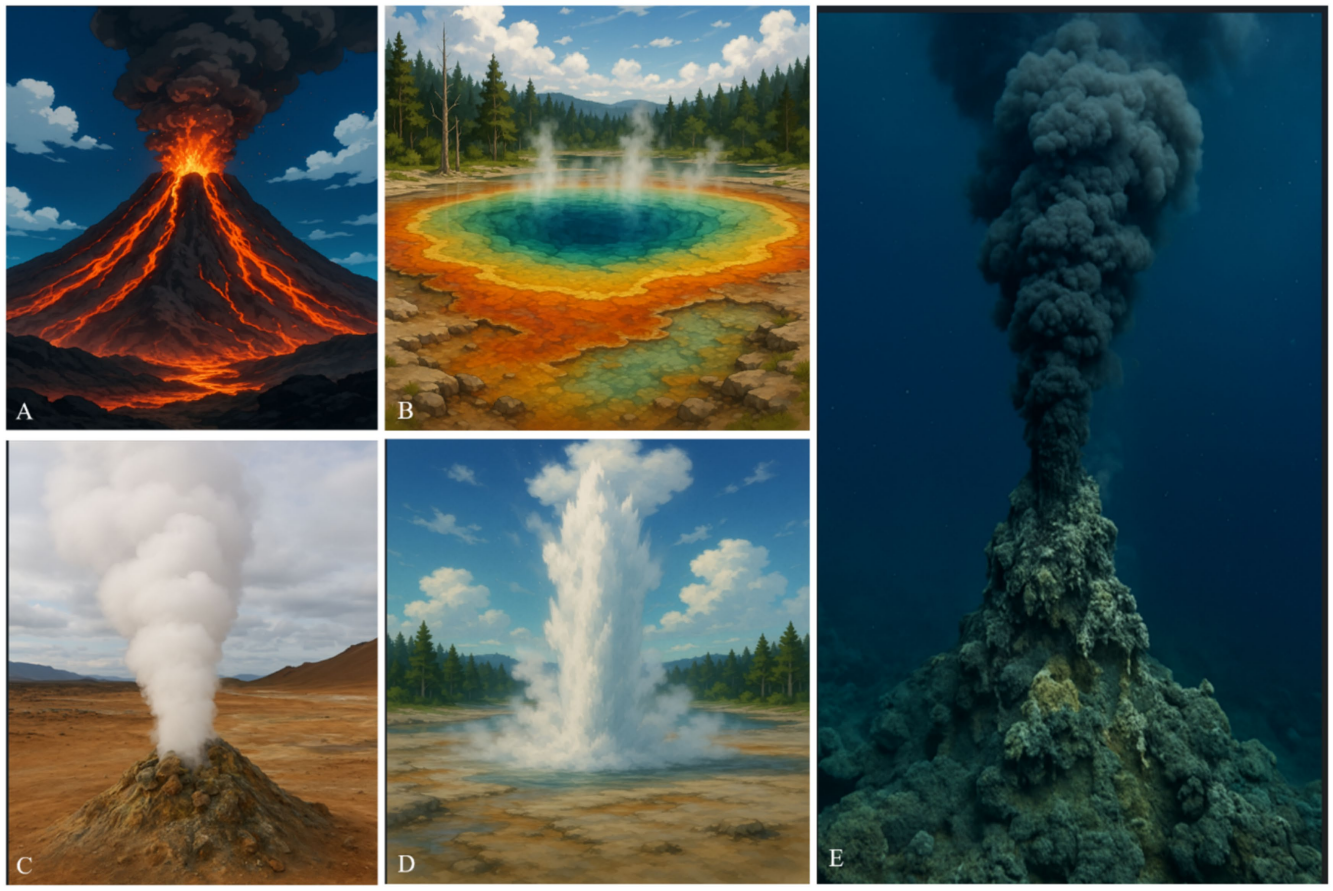
Images of typical hot environments on Earth. **(A)** Volcano—a site of molten lava and extreme heat where thermophiles may inhabit surrounding soils; **(B)** Hot spring—naturally heated water bodies rich in minerals that support diverse thermophilic microbial communities; **(C)** Fumarole—openings in the Earth’s crust emitting steam and gases, providing high-temperature niches; **(D)** Geyser—periodically erupting hot water and steam, creating fluctuating thermal environments; **(E)** Hydrothermal vent—underwater fissures releasing mineral-rich, superheated water, harboring unique thermophilic and hyperthermophilic organisms. All images were generated with the help of OpenAI (free version).

A variety of thermal environments, natural as well as man-made, exist on earth that are populated by the thermophiles. These include volcanic and geothermal areas with temperatures often greater than boiling of water. Themophiles have been studied from hot springs, geysers, volcanoes and deep-sea hydrothermal vents (Figure 2). Thermal environments may originate as a result of solar heating, geothermal activity, intense radiation and combustion processes. Temperatures in solar heated soils and combustion processes can reach up to 60°–70 °C. Thermophiles can also colonize the microenvironments where the temperature is high due to the biological activity, such as compost heaps, saw dust, and the coal refuse piles. Thermophiles are also found in domestic and industrial hot water systems and industrial processes with high temperature. Geothermally heated environments are known to host some of the most remarkable thermophiles and hyperthermophiles (Seckbach, 2013). Although these habitats differ in geological origin, they share physicochemical stresses-elevated temperature, steep thermal gradients, low oxygen availability, and, in many cases, extreme pH or high concentrations of sulfur and metals-that exert strong selective pressure on microbial communities.

Hot springs in volcanic areas may reach high temperatures near boiling point (e.g., above 60 °C). Steam vents (fumaroles) can reach much higher temperatures (up to ~400 °C) due to magmatic heating. In undersea hydrothermal vents (e.g., at seamounts or mid-ocean ridges), fluid temperatures have been measured in the range of 300–400 °C (Barreyre et al., 2025; Schrenk et al., 2013).

Thermophiles have been extensively studied in hot springs and other thermal environments worldwide, encompassing a wide range of temperature conditions. Notable geothermal sites across different continents, including Yellowstone National Park in the USA, Iceland’s geothermal fields, Japan’s solfataric areas, and diverse locations in India such as Bakreshwar (West Bengal), Manikaran (Himachal Pradesh), and Soldhar and Ringigad (Uttarakhand), have yielded significant insights into thermophilic microorganisms (Table 1). These studies highlight the global distribution and diversity of thermophiles thriving in both low- and high-temperature thermal habitats.

**Table 1:** Representative microbiota inhabiting diverse high-temperature environments.

S. No.	Location	Some dominant microbial species/groups	Environmental conditions	Reference
1	Geysers and Hot Springs, Yellowstone NP, USA	*Thermus aquaticus*	~70–75 °C, neutral pH	Brock and Freeze (1969)
2	Agnikunda, India	gamma-*Proteobacteria*, Cyanobacteria, and green nonsulfur	66–69 °C and 9.1–9.3 pH	Ghosh et al. (2003)
3	Soldhar and Ringigad, India	Soldhar: *Proteobacteria*, *Deinococcus*-*Thermus*, *Aquificae*; *Pyrobaculum* (archaeal genus); culturable species of *Bacillus*, *Geobacillus* and *Paenibacillus*; cyanobacterial mats, yeast, and many cultures in VBNC (viable but not culturable) state	90–95 °C at Soldhar, and 75–80 °C at Ringigad, 8.2 pH at Soldhar and 8.1 pH at Ringigad	Kumar et al. (2004), Pandey et al. (2015) and Sharma et al. (2009, 2015, 2017)
4	Geothermal Fields, Tengchong County, China	*Aquificae*, *Crenarchaeota*, *Candidatus Nitrosocaldus yellowstonii*	55.1–93.6 °C and 2.5–9.4 pH	Hou et al. (2013)
5	Kamchatka Thermal Springs, Russia	*Thaumarchaeota*, *Thermotogae*, *Proteobacteria*, others	thermoacidophilic spring (70 °C, pH 3.5–4); thermophilic spring (81 °C, pH 7.2–7.4)	Wemheuer et al. (2013)
6	Los Azufres Geothermal Field, Mexico	*Rhodobacter*, *Acidithiobacillus*, *Thiomonas*, *Desulfurella*, *Thermodesulfobiu*	27–87 °C and 1–3 pH	Brito et al. (2014)
7	Inferno Crater Lake, New Zealand	Sulfur-oxidizing archaeal groups (e.g., like Sulfolobus spp.)	30–80 °C (cyclic); ~ 2.0 pH	Ward et al. (2017)
8	Solfatara Crater, Southern Italy	*Thermoplasma*, *Ferroplasma*, *Acidithiobacillus*	Up to 70 °C and 2.2 pH (very acidic)	Crognale et al. (2018)
9	Mud Volcano (Urania Basin, Mediterranean Sea)	Sulfur cycling and halophilic archaea at different layers	Upto 55 °C	Lazar et al. (2022)
10	Hot water spring clusters in Sri Lanka	*Proteobacteria, Actinobacteria, Firmicutes, [Thermi],* and *Cyanobacteria*.	surface 36–59 °C, and 6.25–8.35 pH	Rupasinghe et al. (2022)
11	Taupō Volcanic Zone (New Zealand)	Proteobacteria, Euryarchaeota, Crenarchaeota	17.5–92.9 °C and 2.0–7.5 pH	Sriaporn et al. (2023)
12	Chumathang geothermal spring, Ladakh, India	bacterial phyla 50, and 42.86% in water and soil samples, respectively; 9.62 and 7.94% of archaeal phyla in water and soil samples, respectively. Firmicutes and Proteobacteria- most abundant bacterial phyla in water; Proteobacteria and Bacteroidetes- most abundant bacterial phyla in geothermal soil.	25 ± 2°C to 87 ± 2°C and 8.6 pH	Anu et al. (2024)
13	Kuril Islands Hot Springs, Russia	*Cyanobacteriota, Chloroflexota, Aquificota*	40–79 °C; pH 5.7–8.5	Karaseva et al. (2024)
14	North Sikkim, India	*Bacillota* (most abundant overall, flourishing in hot and warm regions); *Pseudomonadota* (thriving in cooler areas); *Actinomycetota*; *Chloroflexota* (flourished in hot and warm regions)	Hot region: 56–65 °C Warm region: 35–37 °C Cold region: 4–7 °C	Kumar et al. (2024)

A key driver shaping thermophilic microbial communities is the interplay of temperature, pH, redox chemistry, and mineral composition. For example, Sulfur rich environments such as Solfatara fields, are acidic hot springs and boiling mud pots. Such distinct geothermal environments are widespread in volcanic zones and referred to as ‘high temperature fields’ (Schwartz, 1980). Similar volcanic regions along with Yellowstone National Park, possess heating water that happens due to the interaction with magma resulting g in the elevated temperatures/boiling of water (Inskeep et al., 2013). In addition, geysers are the thermal environments that produces jets of steam and hot water above the Earth’s surface. The moment water reaches the surface as steam, it known as fumarole; and becomes “mudpots” when mixed with mud and clay (Inskeep et al., 2013). Most thermophiles isolated from hydrothermal vent systems-where the combination of high temperatures and hydrostatic pressure maintains water in a liquid state are anaerobic microorganisms adapted to these extreme conditions (Reysenbach and Shock, 2002; Schrenk et al., 2013).

Thermophilic microorganisms inhabit various undersea hot springs and hydrothermal vent systems. Black smoker vents emit extremely hot, metal-rich fluids that precipitate metal sulfides upon mixing with cold seawater, forming characteristic chimney structures. The thin walls of these chimneys can reach temperatures of 200–400 °C, creating extreme microsites that host highly specialized archaeal lineages adapted to rapid temperature fluctuations and intense metal stress. Hyperthermophiles also colonize the seawater surrounding active seamounts, where volcanic lava is emitted directly onto the seafloor (Seckbach, 2013). Additionally, shallow marine hydrothermal systems support diverse bacterial and archaeal communities, as demonstrated in studies of vents near Vulcano Island (Antranikian et al., 2017). Together, these environments illustrate how temperature interacts with geochemistry to structure thermophilic ecosystems, selecting for organisms with unique physiological and metabolic traits. Understanding these physicochemical drivers is essential for identifying novel thermophiles and their thermostable enzymes with potential industrial relevance.

## 3 Taxonomic and ecological spectrum of thermophiles

Moderate thermophiles consist of diverse taxonomic groups (both prokaryotic and eukaryotic microorganisms) generally have optimum growth between 50 °C and 60 °C temperature ranges (Stetter, 2006; Bender et al., 2022). Phylogenetically, moderate thermophiles often appear closely related to mesophilic organisms, indicating their adaptive capacity to thrive in thermal environments. Recently, thermotolerant bacteria belonging to the genera *Bacillus* and *Paenibacillus* have been isolated from hot springs in the temperate regions of the Indian Himalayas, demonstrating growth across a wide temperature range of 20–80 °C with an optimum near 55 °C (Pandey et al., 2015). These bacteria also exhibited tolerance to a broad pH spectrum (4–14) and produced enzymes active in the thermophilic range, underscoring their industrial potential. Notable examples of moderate thermophiles include the cellulolytic bacterium *Clostridium thermocellum* and the acetogenic bacteria *Moorella thermoacetica* and *Moorella thermoautotrophica* (Leschine, 1995; Drake et al., 2008). Aerobic bacteria such as *Geobacillus kaustophilus*, *G. stearothermophilus*, *G. thermoleovorans*, and *Bacillus halodurans* also fall within this category and have been studied extensively for their thermostable enzymes and biotechnological applications (Nazina et al., 2001; Zeigler, 2014).

Extreme thermophiles, that grow optimally between 60° and 80 °C, are represented in the genera *Bacillus*, *Clostridium*, *Thermoanaerobacter*, *Thermus*, *Fervidobacterium*, and *Thermotoga*. Most of these thermophiles are anaerobic Firmicutes that include cellulolytic (*Caldicellulosiruptor saccharolyticus*), ethanol producing (*Thermoanaerobacterium*), and the acetogenic facultative chemolithoautotrophic (*Thermoanaerobacterium kivui*), and denitrifying (*Ammonifex degensii*) bacteria. The extreme aerobic thermophiles include *Bacillus stearothermophilus* (Firmicutes) and within the Gram-negative genus *Thermus*. The examples of some of the recently reported novel thermophilic species are *Sporolituus thermophilus* (citrate fermenting anaerobic bacterium), *Microaerobacter geothermalis* (microaerophilic, nitrate and nitrite reducing bacterium), *Nautilia abyssi* (the sulfur reducing deep sea bacterium), *Anoxybacillus thermarum* (the thermal mud-inhabiting), and *Caldisericum exile* (anaerobic filamentous bacterium of a novel phylum) (Canganella and Wiegel, 2011; Antranikian et al., 2017). Hyperthermophiles represent some of the most ancient and physiologically extreme lineages in both the domains Bacteria and Archaea. Among bacteria, the genera *Aquifex* and *Thermotoga* are canonical representatives, both occupying basal phylogenetic positions and exhibiting optimal growth temperatures above 80 °C (Huber and Stetter, 2001). Within Archaea, hyperthermophiles are taxonomically diverse and include members of the genera *Pyrodictum*, *Pyrobaculum*, *Thermoproteus*, *Desulfurococcus*, *Sulfolobus*, *Methanopyrus*, *Pyrococcus*, *Thermococcus*, *Methanococcus*, and *Archaeoglobus* (Stetter, 1999; Reysenbach and Shock, 2002). These organisms thrive at optimal temperatures ranging from ~80 to 108 °C, inhabiting environments strongly shaped by geothermal activity. Their most common ecological niches include volcanic solfataric fields, terrestrial hot springs, and, most notably, submarine hydrothermal systems (Huber et al., 2000). Hydrothermal vent environments occur at both shallow and abyssal depths and encompass fumaroles, hot springs, mineral-rich sediments, and deep-sea “black smokers,” where vent fluid temperatures can exceed 350–400 °C. While no life forms persist directly in the hottest fluids, hyperthermophiles colonize peripheral mixing zones where steep thermal and chemical gradients allow biological activity (Baross and Hoffman, 1985; Kelley et al., 2001).

The distribution of hyperthermophilic taxa reflects ecological partitioning across hydrothermal gradients. Genera such as *Pyrococcus*, *Pyrodictum*, *Thermococcus*, *Methanococcus*, *Archaeoglobus*, and *Thermotoga* have been recovered from both shallow and deep-sea vent systems, highlighting their physiological versatility (Stetter, 1999; Huber et al., 2000). By contrast, *Methanopyrus* is often associated with greater depths and higher-pressure regimes (Kurr et al., 1991), whereas *Aquifex* has been isolated primarily from shallow hydrothermal environments (Deckert et al., 1998). Hyperthermophiles are metabolically diverse, with pathways including sulfate and sulfur reduction, nitrate reduction, and iron reduction, reflecting adaptations to energy sources available in geothermal systems (Reysenbach and Shock, 2002). Their metabolic versatility makes them critical drivers of biogeochemical cycling in extreme environments. The known upper temperature limit for life has been repeatedly revised as new isolates emerge. *Pyrolobus fumarii*, a vent archaeon, was long regarded as the most thermophilic organism, with an optimum growth temperature of 106 °C and a maximum near 113 °C (Blöchl et al., 1997). More recently, a strain of *Methanopyrus kandleri* (strain 116), recovered from a deep-sea hydrothermal vent, demonstrated active growth at 122 °C under elevated hydrostatic pressure, setting a new upper boundary for life as currently known (Takai et al., 2008). These findings not only expand our understanding of microbial limits but also inform discussions on the potential for life in extraterrestrial high-temperature environments. Examples of thermophiles belonging to Bacteria and Archaea are given in Table 2.

**Table 2:** Examples of thermophilic microorganisms and their optimal growth temperatures.

Thermophile(s)	Optimal growth temperature (°C)
Moderate thermophiles (50–60 °C)	
*Bacillus acidocaldarius*	50
*Bacillus licheniformis*, *B. tequilensis*	55
*Paenibacillus ehimensis*	55
Extreme thermophiles (60–80 °C)	
*Geobacillus kaustophilus*, *G. stearothermophilus*	65
*Thermus aquaticus*	75
*Thermoanaerobacter ethanolicus*	65
*Clostridium thermosulfurogenes*	60
*Frevidobacterium pennivorans*	75
Hyperthermophiles (80–110°C)	
*Archeoglobus fulgidus*	83
*Aquifex pyrophilus*	85
*Sulfolobus sulfataricus*	88
*Thermotaga maritiana*	90
*Pyrococcus furiosus*	100
*Pyrodictum occultum*	105
*Pyrolobus fumarri*	106

Source: Vieille and Zeikus (2001), Sharma et al. (2009), Seckbach (2013), and Pandey et al. (2015).

A range of thermophilic fungi belonging to Zygomycetes (*Rhizomucor miehei*, *R. pusillus*), Ascomycetes (*Chaetomium thermophile*, *Thermoascus aurantiacus*, *Dactylomyces thermophilus*, *Melanocarpus albomyces*, *Talaromyces thermophilus*, *T. emersonii*, *Thielavia terrestris*), Basidiomycetes (*Phanerochaete chrysosporium*) and Hyphomycetes (*Acremonium alabamensis*, *A. thermophilum*, *Myceliophthora thermophila*, *Thermomyces lanuginosus*, *Scytalidium thermophilum*, *Malbranchea cinnamomea*) have been isolated from composts, soils, nesting materials of birds, wood chips and many other sources (Tansey, 1973; Maheshwari et al., 2000; Salar and Aneja, 2007; Ingersoll, 2023). Some algae such as *Synechococcus* (the cyanobacterial genus) have been found in the thermophilic mat communities growing at temperatures above 60 °C (Klatt et al., 2011; George et al., 2023). The moderately hot runoff channels and pools below 60 °C are found populated by cyanobacterial mats. In these mats, the diazotrophic cyanobacteria such as *Fischerella*, *Calothrix*, and *Pleurocapsa* grow in nitrogen poor waters at lower temperatures, whereas *Synechococcus* and *Phormidium* mats are favored by nitrogen rich waters (Hongmei et al., 2005; George et al., 2023). The protozoa (*Cothuria* sp. *Oxytricha falla*, *Cercosulcifer hamathensis*, *Tetrahymena pyriformis*, *Cyclidium citrullus, Naegleria fowleri*) are also reported to grow at high temperatures (Tarkington and Zufall, 2021; Iyevhobu et al., 2025). Some examples of thermophilic microorganisms are presented in Table 3.

**Table 3:** Model thermophiles used in biotechnological research.

S. No.	Thermophilic microorganism	Optimum temp (°C)	Brief description	Reference
1	*Thermus aquaticus*	~70–75	Thermophilic bacterium, source of Taq DNA polymerase for PCR.	Brock and Freeze (1969)
2	*Thermotoga maritima*	~80	Anaerobic, thermophilic	Huber et al. (1986)
3	*Methanopyrus kandleri*	~98–110	Hyperthermophile, obligate chemolithoautotrophic	Kurr et al. (1991)
4	*Pyrolobus fumarii*	~106	Facultatively aerobic obligate, chemolithoautotroph	Blöchl et al. (1997)
5	*Acidilobus aceticus*	~85	Anaerobic thermoacidophile from volcanic springs in Kamchatka.	Prokofeva et al. (2000)
6	*Pyrococcus horikoshii* OT-3	~95	Hyperthermophilic archaeon, endoglucanase	Ando et al. (2002)
7	*Aquifex aeolicus*	~95	Thermophilic, eubacteria	Purcarea et al. (2003)
8	*Geogemma barossii* (strain 121)	~121	Records the highest known growth temperature for an archaeon	Kashefi and Lovley (2003)
9	*Staphylothermus marinus*	~98	anaerobic, hyperthermophile, Crenarchaeota	Anderson et al. (2009)
10	*Sulfolobus solfataricus*	~80	Hyperthermoacidophilic, crenarchaeon	Ulas et al. (2012)
11	*Candidatus Nitrosocaldus cavascurensis*	~68	Chemolithoautotrophic condition, ammonia oxidizing archaea	Abby et al. (2018)
12	*Sulfolobus acidocaldarius*	~75–80	Thermoacidophilic archaeon	Baes et al. (2020)
13	*Thermococcus bergensis* sp.	~80	Strict anaerobic, Chemo-organotroph, Starch degrading	Birkeland et al. (2021)
14	*Pyrobaculum calidifontis*	~90	facultative anaerobic hyperthermophilic crenarchaeon	Fukuda et al. (2022)
15	*Thermococcus kodakarensis*	~85	Anaerobic marine archaeon from Kōdakarajima island; fermentative growth, model genetic system	Su et al. (2023)
16	*Thermosediminibacter oceani*	65–70	Thermophilic bacterium with oxygen-stable [FeFe]-hydrogenase, showing novel oxygen protection	Ghosh et al. (2025)
	*Tardisphaera*	~55–65	Thermoacidophilic anaerobe from Kamchatka springs; ferments sugars	Prokofeva et al. (2025)
17	*Bacillus thermoamylovorans*	55–65	Thermophilic bacterium used to improve metabolic heat accumulation in composting systems	Wang et al. (2025)

## 4 Molecular phylogeny and the emergence of the three-domain system

Until the 60s, the microorganisms were classified as prokaryotes or eukaryotes. In 1965, it was found that certain molecules could act as evolutionary chronometers. The evolutionary distance between two organisms may be inferred from the differences in the amino acid or nucleotide sequences of homologous macromolecules isolated from both (Woese, 1965). The molecules that were proposed as good evolutionary chronometers are ATPase, the RecA protein, and the rRNAs. These molecules were probably essential even for the most primitive cells (Woese and Fox, 1977). Thus, the changes in the sequence of the genes encoded them allowed the establishment of the evolutionary relationships among different microorganisms. In 1977, Carl Woese proposed 16S rRNA molecule as an optimal prokaryote chronometer that led to the prokaryote phylogenetic tree and the discovery of a new group of microorganisms-the Archaea (Woese and Fox, 1977; Woese et al., 1990). The phylogenetic tree of life, constructed from prokaryote 16S rRNA sequences and eukaryote 18S rRNA sequences, subdivide the living organisms into three Domains namely Bacteria, Archaea and Eukarya (Gribaldo and Brochier-Armanet, 2006; Forterre, 2015). In Bacteria and Archaea, 16S rRNA consists of about 1,500 bases. Hyperthermophiles are found in both the Archaea and Bacteria and occupy the most basal positions of the phylogenetic tree (DiGiacomo et al., 2022). Deeply branching lineages in the phylogenetic tree provide evidence of early divergence events. The separation of Bacteria from the common stem shared by Archaea and Eukarya represents one of the earliest and most basal splits in the tree of life. Short branch lengths indicate relatively low rates of molecular evolution. Unlike the eukaryal domain, the bacterial and archaeal domains contain several lineages that are both basal and slowly evolving. Notably, these lineages are predominantly occupied by hyperthermophiles, which cluster near the root of the universal phylogenetic tree. Among them, anaerobic thermophilic Archaea inhabit some of the most extreme environments and correspond to the deepest, most conserved branches in the tree of life. They are found to often use the substrates that are thought to have been dominant in the primordial terrestrial makeup, indicating that they could have been the first living forms on this planet (DiGiacomo et al., 2022).

In Bacteria, hyperthermophiles are primarily represented by the genera *Thermotoga* and *Aquifex*, which occupy some of the most basal lineages within the domain. Cultured Archaea can be classified into two major phyla: Crenarchaeota and Euryarchaeota. The phylum Crenarchaeota, composed entirely of extreme thermophiles and hyperthermophiles, includes genera that are deeply branching and exhibit short branch lengths in rRNA-based phylogenetic trees. Representative hyperthermophilic genera within Crenarchaeota include *Sulfolobus*, *Desulfurococcus*, *Pyrodictium*, *Thermofilum*, *Thermoproteus*, and *Pyrolobus*. Members of Euryarchaeota are more broadly distributed ecologically, yet the most basal lineages within this phylum also correspond to hyperthermophilic genera, such as *Methanococcus*, *Thermococcus*, *Methanopyrus*, and *Pyrococcus* (de Miguel Bouzas et al., 2006; Stetter, 2006; Canganella and Wiegel, 2011).

## 5 Traditional and advanced approaches for thermophile investigation

Environmental samples can be investigated through culture dependent and culture-independent approach for thermophilic microorganisms. In the culture-dependent methods, the specific microbial colonies can be obtained on solid media following purification through repeated subculturing (Madigan et al., 2019). Pure isolates of bacteria and fungi, typically involves the sequencing of 16S rRNA gene and ITS region, respectively for a preliminary identification (Janda and Abbott, 2007). A polyphasic approach is widely accepted for the specie-level identification which integrates phenotypic traits with genotypic information for more accurate classification (Vandamme et al., 1996). The pure microbial cultures can be preserved in certified microbial culture collections for future ecological and biotechnological studies (Tindall, 2007). When discussing thermophiles, we encounter a range of challenges associated with culturing methods. The solidifying media is unstable at high temperatures that make the process tedious for obtaining pure cultures following the aforementioned method, in addition longer incubation periods are required due to the slow growth of microorganisms (Amann et al., 1995). Above all, the culturing method is highly biased due to the variable requirements of different microbial groups (selective media composition and growth conditions) that provide a less accurate picture of microbial diversity (Staley and Konopka, 1985).

Recent advances have significantly expanded the study of thermophiles. High-pressure microfluidic platforms enable rapid phenotyping of thermophilic archaea under simultaneous high-temperature and high-pressure conditions (e.g., *Thermococcus barophilus*) (Cario et al., 2022). In parallel, cell-free systems derived from *Thermus thermophilus* have been adapted for *in vitro* transcription-translation inside microfluidic droplets, allowing ultrahigh-throughput screening of thermostable enzymes directly (Ribeiro et al., 2024). Additionally, modified iChip cultivation strategies have facilitated the isolation of previously uncultured thermo-tolerant bacteria from hot springs (Zhao et al., 2023). Streamlined discovery pipelines that incorporate modern bioprospecting strategies-such as (ultra)high-throughput screening, microfluidics, and metagenomic mining-have greatly accelerated the identification and functional characterization of thermozymes with industrial potential.

On the other hand, culture-independent methods are advantageous due to molecular techniques such community DNA extraction from environmental samples following PCR amplification of the universal markers. It allows the detection of diverse microbial taxa, purely independent of its culturing requirement (Muyzer et al., 1993). Another method, to digest amplified PCR product with restriction enzymes followed by investigating the banding pattern on electrophoretic gel results in the fingerprinting of the community diversity, referred as amplified ribosomal DNA restriction analysis (ARDRA) (Rameshkumar et al., 2012). The efficiency of such culture independent approaches can be understood by the fact that these methods have found to uncover several taxonomic groups which were previously not detected by any of the culturing methods (Hugenholtz et al., 1998). However, the culture-independent methods are not completely unbiased. Some factors, like DNA extraction efficiency, PCR conditions, variation in rRNA gene copy number etc. strongly influence the outcomes for diversity analysis (Suzuki and Giovannoni, 1996; Polz and Cavanaugh, 1998). Mindful integration of both the approaches is important to achieve the high accuracy is suggested for microbial diversity in such extreme environments (Lagier et al., 2012).

The relative abundance of Archaea and Bacteria in thermal environments was initially studied using culture-based techniques. This led to the assumption that Archaea dominate the thermal environments, probably due to the biases associated to the growth media and culture conditions. With the emergence of application of molecular methods such as slot-blot hybridizations of rRNA utilizing oligonucleotide probes targeting the 16S rRNA of Archaea and Bacteria, it was found that bacteria constituted the major population in thermal environments. Culture independent studies based on the sequencing of 16S ribosomal RNA (rRNA) obtained directly from thermophilic biotopes have revealed the existence of an enormous variety of microorganisms growing at high temperatures that are otherwise not cultivable. Some new methods have been developed for studying the thermophiles such as a novel micromanipulation method that allows the isolation of a single cell from a mixed culture using “optical tweezers.” An isolation strategy that combines *in situ* 16S rRNA sequence analysis and specific whole-cell hybridization, with the isolation of the identified single cell by “optical tweezers” has also been proposed (de Miguel Bouzas et al., 2006). Ferrer et al. (2016) and Berini et al. (2017) followed metagenomic approach for studying bioprospecting success of microbial enzymes.

## 6 Physiological and genetic adaptations underlying thermophile thermotolerance

Thermophilic microorganisms adapt to the thermal environments in which they have to live and survive by virtue of various mechanisms. The cell membrane of thermophiles is made up of saturated fatty acids that provide a hydrophobic environment for the cell (Koga, 2012). This helps to keep the cell rigid enough to live at elevated temperatures. The Archaea, which compose most of the hyperthermophiles, contain lipids linked with ether on the cell wall. This layer is much more heat resistant than a membrane formed of fatty acids. High temperature increases the fluidity of membranes (Jain et al., 2014). To maintain optimal membrane fluidity the cell must adjust the composition of the membrane including the amount and type of lipids. The typical fatty acid bilayer structure of the bacterial cytoplasmic membrane would become disrupted at extreme temperatures while the archaeal monolayer membrane composed of phytanyl chains connected to glycerol with ether links is much more resistant (Řezanka et al., 2023).

DNA, RNA, and protein hold the machinery of the cell and they get affected by temperature. At high temperatures, denaturation of their native structures takes place. Some mechanisms, related to nucleic acids that protect these biomolecules against high temperature have been studied. Methylation at 2’OH increases RNA stability by protecting it from the hydrolysis of phospho-di-ester bonds. Apart from this, they also increase the stability by the accumulation of positively charged compounds (polyamines) or ions (K^+^) against the negative charge of phosphate group in the back bone that helps in increasing the Tm of the nucleic acids (Hori et al., 2018). Monovalent and divalent salts enhance the stability of nucleic acids as these salts screen the negative charges of the phosphate groups, and protect the DNA from depurination and hydrolysis. The G–C pair of nucleic acids is more thermostable than the A–T or A–U pairs because of the additional hydrogen bond. But elevated G + C ratios are not found among thermophilic prokaryotes due to the stability of the chromosomal DNA, although thermostability is correlated with G + C content of their ribosomal and transfer RNAs (Hickey and Singer, 2004). Thermophiles also tolerate high temperature by using increased interactions that non-thermotolerant organisms use, namely, electrostatic, disulfide bridge and hydrophobic interactions.

Thermophiles contain thermostable proteins that can resist denaturation and proteolysis. The specialized proteins (chaperonins) produced by these organisms help in refolding of proteins to their native form, after their denaturation. Temperature also affects the structure and function of proteins. Proteins found in thermophiles are generally stable due to the occurrence of various structural changes. It includes the packing of the proteins, increase in ion-pair content, formation of higher-order oligomers, increase in hydrogen bonds, changes in the hydrophobicity, reduction in the amount of thermolabile amino acids, increase in proline, etc. These peculiar features of thermophiles that occur at physiological and genetical levels make their proteins different from that of mesophiles (Counts et al., 2017; Go et al., 2024).

To ensure the integrity of genetic information and to carry out metabolic functions, protection of DNA, especially in thermal environments, will be essential. There are several mechanisms used by thermophiles to maintain the stability of their DNA at high temperatures that are likely to work in synergy. The DNA of thermophiles produce a particular type of DNA topoisomerase, called reverse DNA gyrase, that introduces positive super coils in the DNA molecule at the expense of ATP. This confers a greater stability and renders to DNA more resistant to thermal denaturation. This results in raising the melting point of the DNA to at least as high as the organism’s maximum temperature for growth. Another mechanism associated to some thermophilic organisms is the involvement of some histone-like proteins that helps to preserve the duplex structure of DNA. Another mechanism known to protect DNA from denaturation is the presence in the cytoplasm of some hyperthermophilic methanogenes of large amounts of cyclic 2,3-potasium-bi-phospho-glycerate, that avoids chemical damage such as DNA depurinization. Although high temperature results in denaturation and chemical modification in DNA, yet the DNA of hyperthermophiles, such as *Pyrococcus furiosus*, is known to be more stable *in vivo* than that of a mesophile such as *Escherichia coli* (Lehmann et al., 2023).

DNA transfer in Bacteria and Archaea seems to have played a major role, with respect to adaptation of thermophiles to high temperatures. There are three main mechanisms associated with transferring of DNA: natural transformation, conjugation and transduction. The natural transformation refers to the uptake of DNA from the external environment, mostly emerging from lysed cells. Incoming DNA can be degraded and/or can be incorporated into the chromosomal DNA. The recipient cell is usually in charge of DNA uptake. On the other hand, conjugation is a more invasive mechanism where the donor has control over the transferred DNA. It requires direct contact between two cells that may not be closely related. Mostly, small plasmids are transferred in this process. Conjugation is considered to be the main mechanism responsible for horizontal gene transfer (HGT). Transduction involves viruses that function as vehicles enabling DNA exchange between closely related species. Transfer of DNA via gene transfer agents (GTAs) which are virus-like elements encoded by the host genome, membrane vesicles and nanotubes, which are cellular protrusions that can bridge neighboring cells, are some other mechanisms that are also discussed for DNA transfer (Rothschild and Mancinelli, 2001; van Wolferen et al., 2013). DNA exchange has been demonstrated to occur in the archaeon *Sulfolobus acidocaldarius* by Grogan (1996).

## 7 Biotechnological and industrial potential of thermozymes

**Figure 3:**
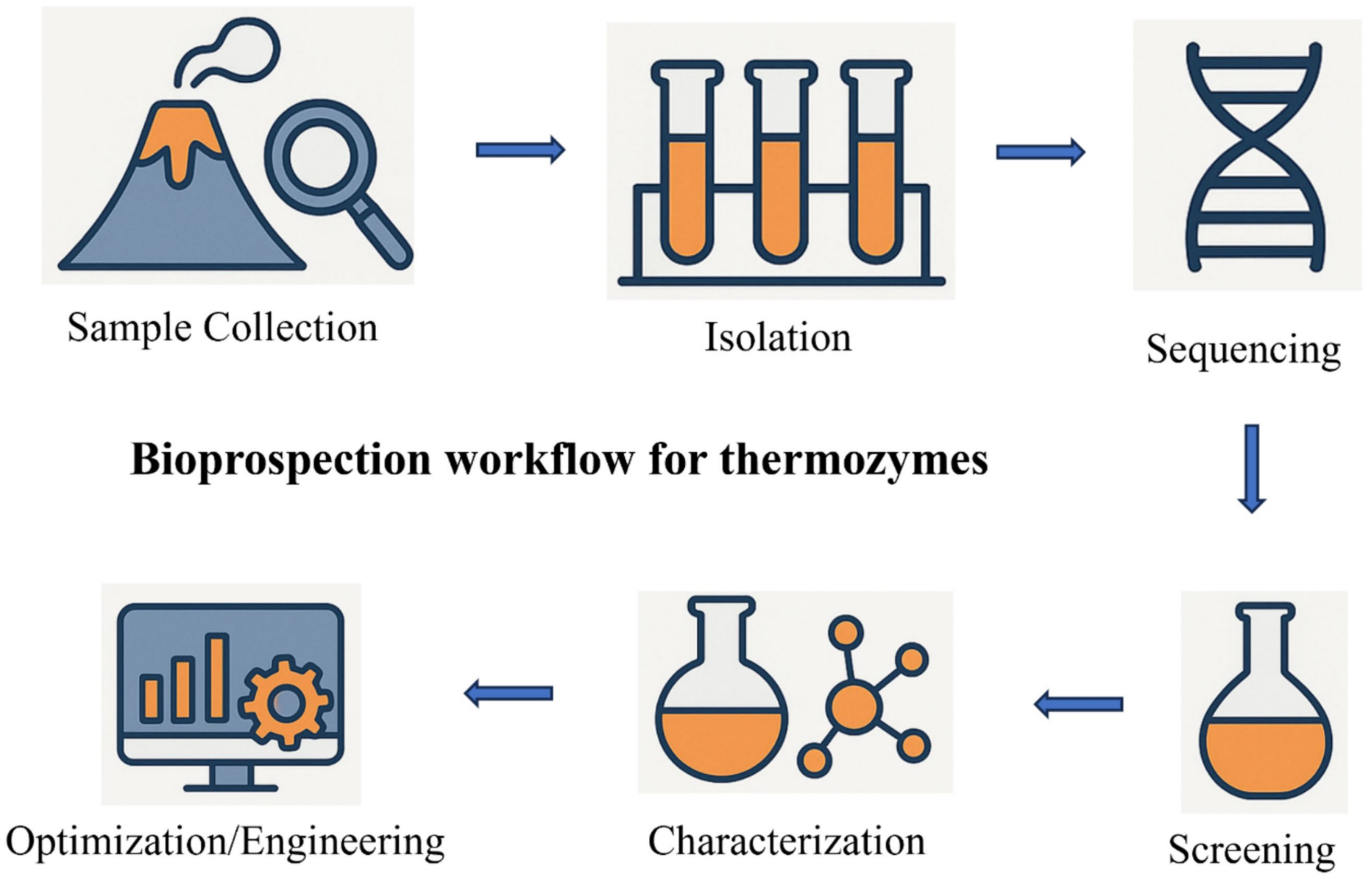
Schematic representation of the bioprospection process for discovering and developing novel enzymes.

The breakthrough research example was set by the discovery of DNA polymerase from a thermophilic bacterium (*Thermus aquaticus*) that was isolated from a hot spring in Yellowstone National Park. The enzyme became the driving ingredient in the polymerase chain reaction that made DNA testing possible and now it supports the billion dollars DNA replication industry. Enzymes from thermophilic microorganisms, often referred to as thermozymes, possess important characteristics such as temperature, chemical, and pH stability. Recent developments in research have shown that thermophiles are a good source of novel catalysts. As illustrated in Figure 3, the bioprospecting workflow typically involves steps from sample collection and isolation of thermophiles to enzyme screening, characterization, and subsequent optimization for industrial applications.

Thermostable polymer-degrading enzymes such as amylases, cellulases, chitinases, lipases, proteases pullulanases, and xylanases are being increasingly recognized for their role in food, chemical, pharmaceutical, paper and pulp, textiles, biorefineries, biofuels, and waste-treatment industries. Overexpression of thermozymes in standard *E. coli* allows the production of much larger quantities of enzymes, which are easy to purify by heat treatment. Most archaeal genes cloned in *E. coli* have been successfully expressed under the control of strong promoters. Once expressed in mesophilic hosts (prokaryotic/eukaryotic), the thermophilic enzymes are easier to purify by heat treatment (Haki and Rakshit, 2003; Koma et al., 2006; Tong et al., 2021). Furthermore, a recent study has demonstrated the application of thermozymes in industrial settings, such as the use of hyperthermoacidic proteases, amylases, and endoglucanases from thermophilic Archaea for efficient removal of thermophilic biofilms from stainless-steel surfaces, highlighting their practical relevance in food and dairy industry sanitation (Nam and Flint, 2023). Table 4 summarizes examples of thermozymes employed in different industrial applications.

**Table 4:** Examples of thermophilic enzymes (thermozymes) and their diverse industrial and biotechnological applications.

S. No.	Source/microbe/strain	Enzyme name	Temperature activity profile	Application	Reference
1	*Sulfolobus solfataricus*	β-galactosidase	Activity reported at 75 °C	Food industries/detergents	Pisani et al. (1990)
2	*Thermus thermophilus* HB8	L-asparaginase	Thermostable at 77 °C (linear kinetics)	Food processing	Pritsa and Kyriakidis (2001)
3	*Thermoanaerobacter thermohydrosulfuricus* SOL1 & *Caldanaerobacter subterraneus* subsp. *tengcongensis*	Lipase	Active between 55 and 90°C	Biocatalysis	Royter et al. (2009)
4	*Geobacillus* sp. strain WSUCF1	β-xylosidase/xylanase cocktail	Optimum activity ~70 °C	Lignocellulosic biomass degradation	Bhalla et al. (2015)
5	*Pyrococcus* sp. M24D13	Nitrilase	Optimum activity ~85 °C	Industrial-scale nitrile hydrolysis or detoxification	Dennett and Blamey (2016)
6	*Thermotoga maritima* (hyperthermophilic bacterium)	Xylanase	Optimum activity ~100 °C	Biodegradation	Yu et al. (2016)
7	*Dictyoglomus turgidum*	β-glucosidase	Optimum ~80 °C	Lignocellulose breakdown, biomass processing	Fusco et al. (2018)
8	*Thermotoga naphthophila* RKU-10	Endo-1,4-β-glucanase (cellulase)	Optimum activity ~90 °C	Biomass/cellulose degradation	Akram and Haq (2020)
9	*Aeribacillus pallidus* BTPS-2 (geothermal spring isolate)	Thermostable α-amylase (algal starch liquefying)	Optimum activity ~70 °C	Starch liquefaction	Timilsina et al. (2020)
10	Thermophilic hot-spring metagenome	Novel multifunctional thermostable α-amylase	Optimum activity ~60 °C	Starch & polysaccharide hydrolysis (also agar, xylan, pectin, cellulose) – useful for food, biofuel, detergent, biomass industries	Bharwad et al. (2023)
11	*Thermus thermophilus* HB27 (as heterologous host)	Mannitol-1-phosphate dehydrogenase (thermostable; from *Thermoanaerobacter kivui*)	Activity reported at 65 °C	Demonstrates thermostable enzyme production in thermophilic host	Kirchner et al. (2023)
12	*Bacillus subtilis* BSP	Thermostable extracellular protease	Optimum activity ~60 °C	useful for detergent, leather, industrial waste treatment	Majeed et al. (2024)
13	*Streptomyces thermodiastaticus* strain TS4	Cellulase	Optimum activity ~60 °C	Lignocellulose breakdown/biomass processing	Waheeb et al. (2024)
14	*Geobacillus* sp.	Thermostable α-amylase	Optimum activity ~75 °C	starch liquefaction	Witasari et al. (2025)

### 7.1 DNA polymerases

The polymerase chain reaction (PCR) is a fundamental biotechnological technique that based on the enzymatic amplification of nucleic acid. The process includes three principal steps, where annealing (the mid step) is very crucial for the successful PCR amplification. Here the thermophilic DNA polymerases form the copies of template DNA (Mullis and Faloona, 1987; Saiki et al., 1988). *Thermus aquaticus*, a thermophilic bacterium was used to obtain the Taq DNA polymerase, functional at high temperatures for PCR. This was the landmark discovery in molecular biology since most of the advanced methods relies on PCR till date, including the gene cloning *Escherichia coli* (Chien et al., 1976; Saiki et al., 1988). After that, a range of thermostable DNA polymerases from various thermophilic and hyperthermophilic archaea were identified and utilized commercially for better results in PCR amplifications. For example, *Pyrococcus furiosus*, (Pfu DNA polymerase), *Thermococcus litoralis* (Vent polymerase), *Pyrococcus woesei* (Pwo polymerase) are being used widely used in PCR based methods (Lundberg et al., 1991; Mattila et al., 1991; Ghasemi et al., 2011). All these enzymes are known to have significant activity even after multiple exposures at high temperatures (98–99 °C) (Chien et al., 1976; Lundberg et al., 1991). Neq2X7 is an engineered fusion polymerase combining a *Nanoarchaeum equitans* DNA polymerase with the Sso7d DNA-binding domain from the thermophile *Sulfolobus solfataricus*, yielding high processivity, inhibitor tolerance, and strong performance on long or GC-rich templates. Although its fidelity is lower than the parental Neq2X enzyme, its robustness and dUTP compatibility make it highly valuable for USER assembly and contamination-resistant diagnostics (Hernández-Rollán et al., 2024).

### 7.2 Molecular cloning

Advancements in protein engineering have provided a solid platform for developing commercially important enzymes with enhanced traits such as stability under extreme conditions (e.g., high temperature, pH variation, presence of oxidizing agents, and organic solvents) (Vieille and Zeikus, 2001; Elleuche et al., 2014). Gene cloning and heterologous expression of thermostable genes in mesophilic hosts like *Escherichia coli* have enabled the large-scale production of engineered enzymes for diverse industrial applications (Nguyen et al., 2004; Kluskens et al., 2005). Recombinant DNA technology has also enabled the enhanced production of enzymes like cellulase in organisms such as *Clostridium* species, which has significant implications in biomass degradation and biofuel industries (Lynd et al., 2002). Moreover, thermophilic microorganisms including *Pyrococcus furiosus*, *Thermococcus kodakarensis*, *Sulfolobus solfataricus*, and *Thermotoga maritima* have emerged as model organisms in both basic research and industrial biotechnology due to their unique enzymatic adaptations to high-temperature environments (Atomi et al., 2012; Hedlund et al., 2015). These thermophiles serve as key systems for understanding molecular adaptations to extreme heat and are invaluable sources of thermostable biocatalysts for various biotechnological applications (Grogan, 1996; Mesbah, 2022). In addition, recently, an RNA ligase from the hyperthermophilic archaeon *Palaeococcus pacificus* (PpaRnl) was characterized; it showed a melting temperature (Tₘ) of ~94 °C and strong adenylation activity across a range of oligonucleotide substrates (Rousseau et al., 2024).

### 7.3 Thermostable enzymes of commercial importance

#### 7.3.1 Amylases

α-Amylases, β-amylases, and pullulanases are some of the enzymes require to degrade the polysaccharide starch (Gupta et al., 2003). These enzymes are present in abundance in thermophilic microorganisms. *Bacillus subtilis*, *B. amyloliquefaciens*, and *B. licheniformis* are some of the identified sources of thermostable α-amylases while *Pyrococcus woesei*, *P. furiosus*, *Thermococcus profundus*, and *T. hydrothermalis* are known to produce hyperthermophilic α-amylases having optimal temperature ~100 °C (Vieille and Zeikus, 2001; Van Der Maarel et al., 2002). The recombinant thermostable glucoamylases have been obtained from *Sulfolobus solfataricus* along with expression in *E. coli*, optimally active at ~ 90 °C and pH 5.5–6.0 (Kim et al., 2004).

#### 7.3.2 Cellulases

The most abundant organic polymer: Cellulose (β-1,4-glucan) is degraded by an enzyme system of cellulases (endoglucanases, exoglucanases, and β-glucosidases) that hydrolyze cellulose in glucose (Bhat, 2000; Wilson, 2009). Identified thermostable cellulases are important in pharmaceuticals, textiles, detergents, and waste treatment (Bhat, 2000). *Pyrococcus furiosus*, *P. horikoshii*, *Sulfolobus solfataricus*, and *Thermotoga maritima* have been recognized as potential source of hyperthermophilic cellulases (Doi and Kosugi, 2004; Ajeje et al., 2021). These enzymes often show optimal activity at high temperatures, making them ideal for industrial processes requiring thermal stability.

#### 7.3.3 Chitinases

Chitinases hydrolyze chitin (β-1,4-linked polymer of N-acetylglucosamine (chitin), widely present in fungal cell walls, insect exoskeletons, and crustacean shells, is the second most abundant natural polymer after cellulose) through endo- and exo-acting enzymes such as chitinase A, chitinase B, and N-acetyl-D-glucosaminidase (Gooday, 1990; Synstad et al., 2008). Chitinases active at elevated temeperatures have been obtained from thermophilic strains including *Bacillus licheniformis*, *B. stearothermophilus*, *Streptomyces thermoviolaceus*, and *Thermococcus chitinophagus* (Mathew et al., 2021), useful for the bioconversion and industrial processes based on chitinous waste.

#### 7.3.4 Lipases

Lipases hydrolyse and synthesize the long chain acylglycerols, having industrial applications in focusing on detergents, food modification, pulp and paper processing, and pharmaceuticals (Jaeger and Reetz, 1998; Sharma et al., 2001). Hyperthermophilic archaea such as *Pyrobaculum calidifontis* and *Pyrococcus furiosus* are the source of thermostable lipases, recombinantly expressed in *E. coli* (Branco et al., 2010; Mehboob et al., 2020). Such enzymes are active under harsh environmental conditions, including high temperature and solvent exposure.

#### 7.3.5 Proteases

Proteases are widely used across various industries including food processing, leather treatment, pharmaceuticals, and textiles. These enzymes are broadly classified as endopeptidases and exopeptidases based on their catalytic mechanisms (Gupta et al., 2002). Thermostable proteases have been isolated from hyperthermophilic bacteria such as *Thermotoga maritima*, *T. aggregans*, and *T. celer*, as well as from archaea like *Pyrococcus* species. These enzymes exhibit exceptional stability and resistance to detergents under extreme conditions, making them highly valuable for industrial applications (Pantazaki et al., 2002; Elleuche et al., 2014). Furthermore, an intracellular protease from *Pyrococcus furiosus* was cloned and expressed in *Escherichia coli*, confirming its thermostability and biotechnological relevance (Bauer et al., 1996). Recently, Uehara et al. (2025) improved the heterologous expression of a hyperthermophilic subtilisin-like protease by using its propeptide in *E. coli*, significantly increasing yield while maintaining activity at high temperature (Uehara et al., 2025).

Enzymes sourced from thermophiles offer several advantages, including high thermal stability, resistance to denaturation, and the ability to function under extreme industrial conditions, making them ideal for applications in biotechnology, pharmaceuticals, and biofuel production. They often exhibit unique substrate specificities and prolonged shelf life compared to mesophilic enzymes. However, there are some limitations, such as difficulties in large-scale production, potential higher costs, and challenges in optimizing activity under mesophilic conditions. Additionally, eukaryotic thermophiles, such as thermophilic fungi, remain less explored, which limits the diversity of available enzymes. Overall, the benefits often outweigh the challenges, especially for applications requiring robust enzymatic performance under extreme conditions.

## 8 The scientific potential and socio-economic dimensions of natural thermal sites

### 8.1 Genomics and systems biology-based approaches to overcome challenges in industrial

#### 8.1.1 Processes involving thermophiles

Thermophiles have garnered unavoidable interest in industrial biotechnology due to their thriving ability at higher temperatures (~100 °C). The extremozymes are remarkably active at comparatively high temperatures, providing an advantage of minimal contamination risks, accelerated reaction rates, and enhanced substrate accessibility through modified solubility (Zeldes et al., 2015; Mesbah, 2022). Still, there are some hurdles toward the efficient use in industrial processes, including depressed growth rates, optimization of active metabolic pathways, and the narrowness of genetic tools (Turner et al., 2007; Millgaard et al., 2025). Approaches based on genomics and system biology offer comprehensive strategies to address these challenges by leveraging high-throughput sequencing, computational modeling, and synthetic biology to improve the performance and utility of thermophilic organisms in industrial applications (Zheng et al., 2019; Ye et al., 2023).

Genomics forms the basis for exploiting thermophiles in industrial processes by revealing their complete genetic and regulatory architecture. The advancement of next-generation sequencing has speed up the process, cost-effective sequencing of thermophilic genomes, yielding high-quality insights into the metabolic potential, thermostability mechanisms, enzymatic profiles and stress responses. For instance, the sequence analysis and multi-omics-based investigations of *Thermotoga maritima* unravel its aligned genome architecture, transcriptional elements, promoter and features of ribosome-binding site, and the strong interaction between mRNA and protein expression, enhanced understanding of its hyperthermophilic trait (Latif et al., 2013). For *Geobacillus* species, the genome sequencing of *G. thermoleovorans* G4, revealed diverse carbohydrate-degrading enzymes—such as amylases, cellulases, pectinases and pullulanases, indicating the strong industrial utility in biomass conversion at high-temperature environments (Ticona et al., 2025).

In case of system biology, omics technology (metagenomics, transcriptomics, proteomics and metabolomics) enables detail insights into thermophilic cellular responses and pathway bottlenecks (Trauger et al., 2008; Go et al., 2024). The algorithms integrated with the gene sequence information through omics, result in the powerful approaches to predict the metabolism of thermophiles are genome-scale metabolic models (GEMs) and constraint-based tools like flux balance analysis (FBA) (Gautam and Xu, 2021; Mol et al., 2021). These modeling approaches provide predictions for optimal conditions, media formulations, and identification of gene knockout targets to manipulate the final product. The simulations of the cellular response to the given conditions reduce the cost and time of the optimization process. The upgraded computational toolbox (MetaboTools) enables the integration of metabolomics with metabolic models to study the phenotypes and metabolic status (Aurich et al., 2016). However, algorithmic toolkits like COMETS support the extension of dynamic FBA to spatial and evolutionary contexts, further enriching design strategies for microbial systems in industrial environments (Dukovski et al., 2021).

Thermophiles, the colonizers of extremely hot environments have allowed us to widen out horizon toward the life associated to early Earth conditions and also extraterrestrial life. If we talk about, terrestrial ecosystems with higher temperatures, biogeochemical cycling is one of the significant roles of thermophiles. The crucial transformation of sulfur, nitrogen and carbon is included in such processes. For example, Sulfolobus species oxidize elemental sulfur to sulfuric acid at temperatures around 70 °C and acidic pH, exerting a profound influence on local pH levels and mineral compositions of geothermal soils (Shivvers and Brock, 1973). In addition, hyperthermophiles such as *Thermotoga maritima*, known for fermenting organic substrates, production of hydrogen gas and organic acids like acetate and lactate. Syntrophic microbial consortia is supported by such small metabolites and significantly contribute in extreme ecosystems (Counts et al., 2017).

Thermophiles also offer valuable insights into the early evolution of life. Molecular phylogenetics and the thermostability of ancestral enzymes suggest that the Last Universal Common Ancestor (LUCA) may have been a thermophile or hyperthermophile (Di Giulio, 2003; Akanuma et al., 2013). Thus, studying modern thermophiles is akin to probing ancient survival strategies and metabolic architectures that allowed life to thrive under primordial, harsh conditions.

### 8.2 Astrobiological significance of thermophiles

Habitability of extraterrestrial environments is being tried to be understood by the astrobiologists through the investigations on model organisms, one of them are thermophiles. Thermophiles are adapted to survive in extremities of radiation, temperature, pressure, and chemical on Earth, such unique abilities make them a promising candidate for analogs to life forms exist in extraterrestrial environments, planets and moons (Carré et al., 2022). Based on astrobiological studies, some of the celestial bodies such as Europa, Enceladus and Mars are hypothesized to harbor subsurface heat sources, tidal or geothermal. Such energies when intertwined with or combined with the available liquid in the environment and chemical gradients, creates niches potentially hospitable for life, mainly for thermophilic and chemolithotrophic microorganisms (Shock and Canovas, 2010; Vance et al., 2018). In addition, hydrothermal vent ecosystems colonized by thermophiles and hyperthermophiles are one of the potential analogs for such extraterrestrial habitats. In such niches, the inhabiting microbial communities obtain energy for metabolic processes from oxidation of sulfur and assimilate hydrogen, unlike sunlight-reliant microbes (Jannasch and Mottl, 1985). The discovery of plumes erupting from Enceladus, containing water vapor, organic compounds, and evidence of ongoing hydrothermal activity, bolsters the hypothesis that similar habitats might exist beneath its icy crust (Waite et al., 2017). The extreme pressure, temperature, and chemical conditions of such environments parallel the natural niches of thermophiles on Earth, suggesting these organisms or similar life forms could survive and thrive beyond our planet.

In addition to life in extremes, Mars faces extremely harsh radiation and desiccating environmental conditions at the surface whereas icy moons probably possess high salinity brines. The robust DNA repair mechanisms, protein thermostability, and specialized membrane lipids in thermophiles contribute to their survival under these stresses (Rothschild and Mancinelli, 2001). Investigating thermophilic extremophiles supports insights into the unavoidable molecular adaptations for life under such stresses, informing models about how life possibly persists in environments outside Earth. In addition to ecological and evolutionary significance, thermophilic enzymes, have practical applications in astrobiology. Intrinsic stability under elevated temperatures and chemical boundaries makes them preferred candidate for in-situ resource utilization (ISRU) strategies for long-duration missions (Elleuche et al., 2014). For instance, extremozymes can be utilized in the bioleaching process from extraterrestrial regolith or enable waste treatment and biosensing in extreme planetary environments (Demirjian et al., 2001). Such biotechnology-based approaches have the potential to reduce payload mass and improve mission sustainability through on-site generation of materials and diagnostics.

Another contribution of thermophiles in the respective research area is associated with their unique functional traits. Metabolic activities of thermophiles possess distinct pattern (isotopic ratios, organic molecules, and bioaltered minerals) that are being considered as promising detectable markers of life/biological activities (Summons et al., 2011). Missions such as Mars 2020 (Perseverance rover) and the upcoming Europa Clipper are designed with instrumentation capable of identifying such biosignatures, informed by our understanding of extreme biology (Grotzinger et al., 2014; Dannenmann et al., 2023).

Finally, we can say that, thermophiles can be considered as indispensable models for astrobiology, providing insights into life’s limits, potential habitats beyond Earth, and tools for detecting extraterrestrial life. Unique adaptations of thermophiles make it a suitable candidate to extreme environments offer a blueprint for life’s persistence and evolution under conditions previously thought inhospitable, thus expanding the scope of astrobiological exploration.

**Figure 4:**
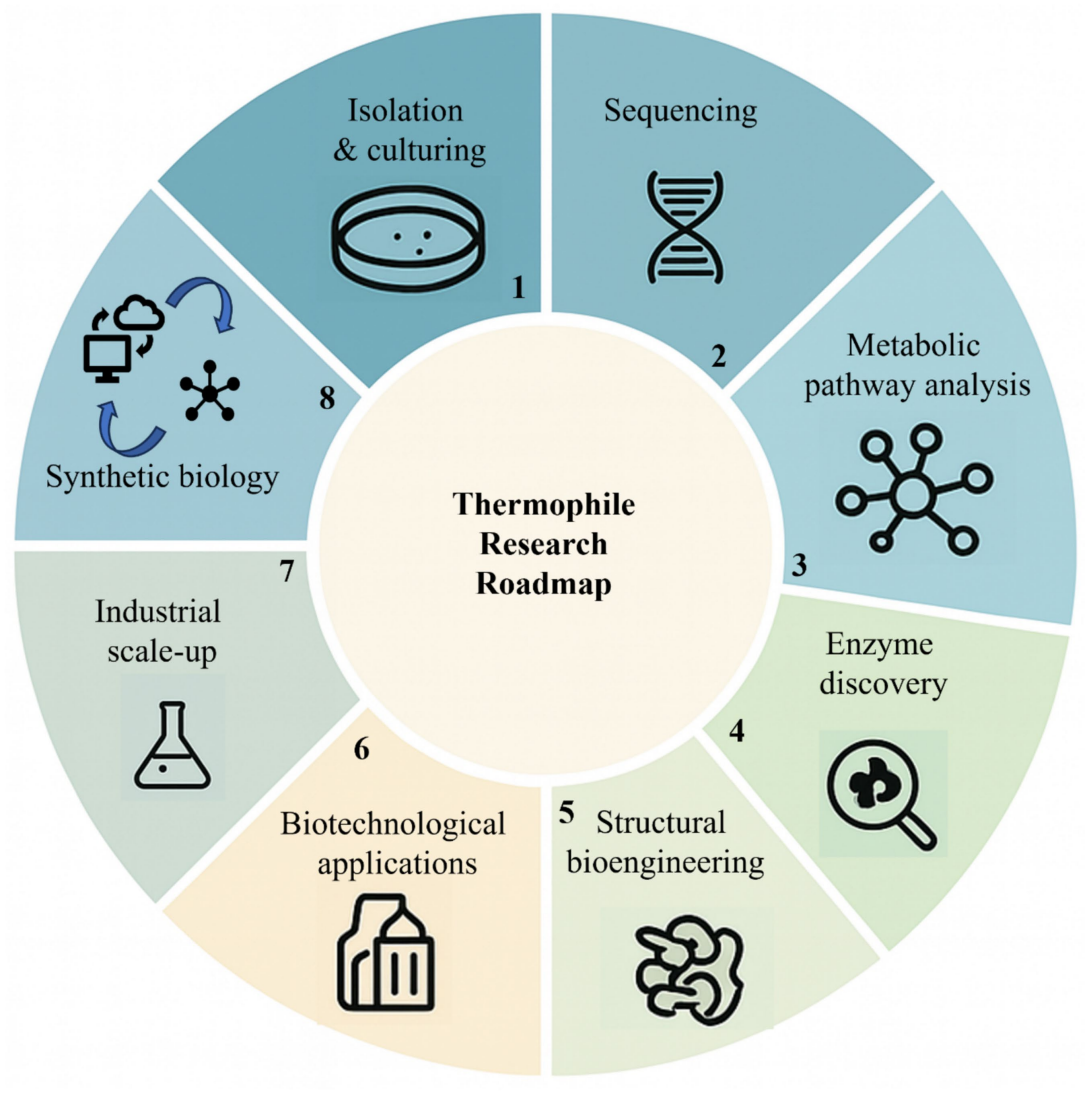
Roadmap for current and future studies on thermophiles and thermozymes. This roadmap emphasizes both fundamental studies and applied research for advancing the utilization of thermophiles and their enzymes in biotechnology.

A roadmap of current and future research on thermophiles, as shown in Figure 4, is highlighted by the integration of genomics, systems biology, and biotechnological strategies to harness thermozymes for industrial and scientific applications.

### 8.3 Preservation and sustainable use of thermal environments

Different thermal environments (hot springs, geothermal vents, and hydrothermal systems) inhabit diverse and unique microbial ecosystems dominated by thermophilic microorganisms. These niches are rich reservoirs of biodiversity and contribute significantly in biogeochemical cycling/associated ecological processes (Inskeep et al., 2013; Bender et al., 2022). In addition, thermophiles are the abundant mines of thermostable enzymes and novel metabolites/biomolecules that possess vast biotechnological potential (Elleuche et al., 2014). To maintain the ecological balance and for sustainable scientific and industrial advancements, the preservation of thermal habitats is necessary. However, thermal environments face increasing threats from human activities including tourism, geothermal energy extraction, mining, and pollution. The unregulated extraction of geothermal fluid can manipulate temperature and chemical profiles, disturbing the unique microbial communities adapted to stable conditions (Nordstrom and Alpers, 1999). The survival of endemic thermophiles may be threatened by foreign interventions, including the introduction of pollutants and other anthropogenic activities (Pointing and Belnap, 2012; Inskeep et al., 2013).

Sustainable management of these environments requires a multidisciplinary approach that integrates environmental impact assessments, regulated resource use, and robust conservation policies. For example, geothermal power development can supply renewable energy but needs strict monitoring and regulation to reduce ecological damage (Lund et al., 2011). Efficient policies for tourism must be implemented with strict rules and regulations to protect fragile microbial mats and geologic features. Ethics should be followed strongly for the sustainable sampling protocols to preserve the original microbial diversity (Alves et al., 2018). Collaborative efforts between scientists, local communities, policymakers, and industries are vital for the effective conservation of thermal habitats. Public awareness campaigns and educational initiatives highlighting the ecological and economic importance of these systems can foster stewardship and encourage responsible use. Several international and national frameworks provide practical steps for protecting these ecosystems. For example, Yellowstone National Park in the USA, a UNESCO World Heritage Site, has established the Geyser Protection Area to restrict geothermal drilling and groundwater extraction, preserving geyser activity and microbial communities (UNESCO, 2025). By balancing conservation with sustainable use, thermal environments can continue to offer vital ecological services, scientific insights, and biotechnological resources for future generation.

## 9 Conclusion

The research areas relevant to thermophilic microorganisms are expanding across diverse fields. Microbiome investigations through culture-independent methods continue to unravel hidden functional capabilities within microbial communities. Although thermophilic fungi-the only eukaryotic lineage capable of thriving at elevated temperatures-have long been proposed as a rich source of thermostable enzymes, their enzyme repertoires remain underrepresented in public databases and in systematic enzyme discovery efforts compared to bacterial and archaeal thermophiles (Maheshwari et al., 2000; Mohammad et al., 2023). Promoting systematic research on thermozymes from thermophilic fungi could uncover novel enzymes with unique properties, offering significant advantages for industrial and pharmaceutical applications. Ongoing improvements in cultivation techniques and molecular biology are essential for advancing biotechnological research. Future investigations could focus on enhancing the thermal stability and catalytic efficiency of thermozymes through protein engineering and bioinformatics, thereby broadening their industrial and pharmaceutical applications. For example, the thermal stability and catalytic efficiency of thermozymes can be enhanced through protein engineering and bioinformatics tools, expanding their applications in industrial processes and pharmaceutical biotransformations. This will facilitate the discovery and deployment of new thermostable enzymes to meet growing demands in industry and clinical sectors.

In addition, adaptation strategies, including mechanisms of DNA transfer under extreme conditions and structural and functional adaptations in these organisms, remain important topics in fundamental research. Finally, the conservation of natural thermal environments requires focused attention from environmental managers. Initiatives such as the protection of the Geyser Basins of Yellowstone National Park under the “Geyser Protection Area” (Barrick, 2010) serve as examples of such efforts. Overall, a roadmap for future research should focus on: (i) integrating advanced omics approaches with improved cultivation strategies, (ii) engineering novel thermozymes for diverse industrial applications, (iii) exploring fundamental adaptive mechanisms in thermophiles, and (iv) conserving thermophilic ecosystems to enable sustainable utilization and study of these microorganisms.

## Footnotes

1 https://serc.carleton.edu/microbelife/extreme/extremeheat/index.html
